# Relationship between subjective well-being and cognitive performance in older adults

**DOI:** 10.1590/1980-5764-DN-2025-0452

**Published:** 2026-06-15

**Authors:** Sabrina Aparecida da Silva, Tiago Nascimento Ordonez, Gabriela dos Santos, Henrique Salmazo da Silva, Maria Antônia Antunes de Souza, Diana dos Santos Bacelar, Laydiane Alves Costa, Rosa Yuka Sato Chubaci, Beatriz Aparecida Ozello Gutierrez, Allan Gustavo Bregola, Alexsandro Roberto Nascimento Ordonez, Sonia Maria Dozzi Brucki, Thais Bento Lima da Silva

**Affiliations:** 1 Universidade de São Paulo, Escola de Artes, Ciências e Humanidades, Departamento de Gerontologia, São Paulo SP, Brazil.; 2 Universidade Federal do Recôncavo da Bahia, Santo Antônio de Jesus BA, Brazil.; 3 Central London Community Healthcare NHS Trust, London, United Kingdom.; 4 Universidade Federal do ABC, São Bernardo do Campo SP, Brazil.; 5 Universidade de São Paulo, Faculdade de Medicina, Grupo de Neurologia Cognitiva e do Comportamento, São Paulo SP, Brazil.

**Keywords:** Personal Satisfaction, Cognition, Aged, Satisfação Pessoal, Cognição, Idoso

## Abstract

The study of subjective well-being (SWB) is fundamental to positive psychology, being considered a potential protective factor for health and the maintenance of cognitive reserve during aging. Despite international evidence, there are gaps in the Brazilian literature regarding how specific components of SWB relate to different cognitive domains.

**Objective:**

To analyze the relationship between subjective well-being and cognitive performance in cognitively healthy Brazilian older adults.

**Methods:**

A longitudinal study was conducted with 183 older adults (mean age 67.3 years). Scales for General Life Satisfaction (SGV), Domain-Referenced Life Satisfaction (SVRD), and the Positive and Negative Affect Scale were applied. Cognitive performance was assessed using the Addenbrooke’s Cognitive Examination-Revised (ACE-R) and the Mini-Mental State Examination (MMSE). Data were analyzed via Spearman’s correlation matrix.

**Results:**

The sample showed a female predominance and high educational level (mean of 17 years). Participants exhibited high levels of SWB and good cognitive performance. General life satisfaction correlated positively with the MMSE and the attention/orientation domains of the ACE-R. Satisfaction with mental capacity (SVRD_Mental) showed a significant correlation with global performance in both cognitive tests. However, no significant correlations were found between affect states (positive or negative) and cognition in this sample.

**Conclusion:**

Healthy, highly educated older adults exhibit high levels of subjective well-being and satisfactory cognitive performance. The results reinforce the association between life satisfaction and cognitive reserve, suggesting that SWB may act as a marker of successful aging.

## RESUMO

O estudo do bem-estar subjetivo (BES) é fundamental na psicologia positiva, sendo considerado um potencial fator protetor para a saúde e manutenção da reserva cognitiva durante o envelhecimento. Apesar das evidências internacionais, há lacunas na literatura brasileira sobre como os componentes específicos do BES se relacionam com diferentes domínios cognitivos.

**Objetivo:**

Analisar a relação entre o bem-estar subjetivo e o desempenho cognitivo em idosos brasileiros cognitivamente saudáveis.

**Métodos:**

Estudo longitudinal realizado com 183 pessoas idosas (média de 67,3 anos de idade). Foram aplicadas escalas de Satisfação Geral com a Vida (SGV), Satisfação com a Vida Referenciada a Domínios (SVRD) e a Escala de Ânimo Positivo e Negativo. O desempenho cognitivo foi avaliado através do *Addenbrooke’s Cognitive Examination-Revised*(ACE-R) e do Miniexame do Estado Mental (MEEM). Os dados foram analisados via matriz de correlação de *Spearman*.

**Resultados:**

A amostra apresentou predominância feminina e alta escolaridade (média de 17 anos). Os participantes exibiram altos níveis de BES e bom desempenho cognitivo. A satisfação geral com a vida correlacionou-se positivamente com o MEEM e domínios de atenção/orientação do ACE-R. A satisfação com a capacidade mental (SVRD_Mental) apresentou correlação significativa com o desempenho global em ambos os testes cognitivos. Contudo, não foram encontradas correlações significativas entre os estados de afeto (positivo ou negativo) e a cognição nesta amostra.

**Conclusão:**

Idosos saudáveis e com alta escolaridade apresentam níveis elevados de bem-estar subjetivo e desempenho cognitivo satisfatório. Os resultados reforçam a associação entre a satisfação com a vida e a reserva cognitiva, sugerindo que o BES pode atuar como um marcador de envelhecimento bem-sucedido.

## INTRODUCTION

In the field of developmental psychology, the study of well-being has become central to the growth of positive psychology. This approach seeks to expand scientific focus beyond psychological pathology, to encompass protective aspects that are valuable for human subjectivity^1^. In this context, well-being (WB) can be conceptualized from different perspectives, with an emphasis on subjective well-being (SWB) and psychological well-being (PWB)^2^.

The concept of SWB was introduced by Diener^3^, and denotes the assessment individuals make of their own life, encompassing both affective and cognitive aspects. According to the author, SWB comprises three main dimensions: life satisfaction, positive affect, and negative affect. Satisfaction with life represents the overall assessment a person has of their life trajectory, whereas positive and negative affects reflect the predominance of pleasurable and unpleasant emotions in everyday life, respectively.

According to Anglim^2^, high SWB is characterized by a high level of satisfaction with life, involving factors such as health, memory, finances and social support, besides a predominance of positive affects and low incidence of negative affects. This rating is influenced by cognitive aspects, based on individual standards and beliefs, as well as emotional factors, which reflect everyday experiences and lived events^3^.

Despite the physical, cognitive and social losses that often accompany the aging process, SWB tends to remain stable in late life, at least for the first 12 years after reaching this phase of life^4,5^. In addition, sociodemographic variables such as age, sex, education and income have been widely investigated in this context, given that they can influence levels of SWB^4,6^.

Levels of SWB and PWB have been recognized as important protective factors for health, being associated with lower incidence of chronic diseases, such as hypertension, hypercholesterolemia, diabetes, and sleep disorders, and also with more serious conditions, such as Alzheimer and cardiovascular diseases, including risk of stroke and acute myocardial infarction^7^. Moreover, these types of well-being have been associated with greater engagement in self-care practices and longevity^8,9^.

Evidence indicates that higher levels of SWB are associated with superior cognitive performance across multiple domains, including information processing, learning, memory, and executive functions^10,11^. This relationship may be explained by the involvement of multiple neurobiological systems that integrate affective and cognitive processes. The regulation of the hypothalamic-pituitary-adrenal (HPA) axis appears to play a central role, as adequate cortisol modulation is associated with better functioning of the hippocampus and prefrontal cortex, whereas chronic dysregulation of this axis can impair memory and executive control^12,13^.

Neuroimaging studies have also demonstrated that SWB is related to the organization and connectivity of large-scale neural networks, particularly the default mode, salience, and frontoparietal control networks, which are essential for attention, memory retrieval, and cognitive flexibility^14^. Moreover, monoaminergic neurotransmission involving dopamine and serotonin contributes to the regulation of motivation, mood, and executive functions, reinforcing the link between affective states and cognitive efficiency^15,16^. Positive affective states have also been associated with reduced inflammatory responses and enhanced neuroplasticity, processes that may sustain cognitive resilience and protect against age-related decline. Taken together, these findings suggest that SWB influences distributed neurocognitive mechanisms that support cognitive performance and reserve throughout aging.

Studies have shown that individuals reporting higher SWB or life satisfaction exhibit greater neural efficiency and functional connectivity in regions supporting memory and executive processes, suggesting that positive affect may enhance compensatory network recruitment^17^. Moreover, SWB is positively associated with engagement in cognitively stimulating, social, and physically active behaviors that sustain neuroplasticity and reserve accrual over time^18,19^. These findings highlight SWB as both a marker and a modifiable factor in the maintenance of cognitive reserve, underscoring its relevance for interventions aimed at promoting resilience and delaying cognitive decline in older adults.

Despite growing international evidence, the specific relationships between SWB components and distinct cognitive domains remain insufficiently characterized in Brazil. Psychometric studies have confirmed the tripartite structure of SWB in Brazilian samples and emphasized the importance of examining life satisfaction and affective components separately when investigating correlates and outcomes of well-being^20^. Research involving Brazilian older adults also indicates that participation in educational and social programs, as well as favorable psychosocial conditions, are associated with higher levels of SWB, suggesting potential behavioral pathways through which well-being may contribute to cognitive maintenance^21^. Furthermore, national studies show that SWB is intertwined with functional status, perceived health, and regional sociodemographic contexts, factors that may modulate trajectories of cognitive aging^22^. Cross-sectional evidence demonstrates associations between higher SWB, better self-rated health, and superior functional performance among community-dwelling older adults, while regionally stratified analyses of long-lived cohorts reveal that social support and leisure engagement predict greater life satisfaction in specific regions of Brazil^22,23^.

Overall, these findings highlight a gap in the literature and reinforce the need for longitudinal and analytical studies that investigate the components of SWB in relation to cognitive functioning, particularly within the Brazilian context. Preserving cognitive abilities is of the utmost importance for aging, representing one of the pillars in maintaining independence and promoting quality of life in older age. Adverse factors that compromise health, such as the presence of multimorbidities, can negatively influence levels of life satisfaction. Therefore, the aim of the present study was to analyze the relationship between SWB and cognitive performance in cognitively healthy Brazilian older adults, contributing to the understanding of how affective and cognitive processes interact to support healthy aging.

## METHODS

This is a longitudinal study that draws on the randomized clinical controlled trial entitled *“A eficácia de um programa de estimulação cognitiva com componentes multifatoriais na cognição e em variáveis psicossociais de idosos sem demência e sem depressão*” [*Effectiveness of a program with multifactorial components on cognition and psychosocial variables of older adults with no dementia or depression*], conducted between 2021 and 2024. The present study sample comprised 183 older adults, all of whom were participants in the above-mentioned clinical trial.

The inclusion criteria were individuals with normal performance for age and education on the following instruments: Mini-Mental State Exam (MMSE)^24^, Pfeffer Functional Activities Questionnaire (FAQ score<2)^25^, Geriatric Depression Scale (GDS<5)^26^, and Geriatric Anxiety Inventory (GAI<10)^27^.

Exclusion criteria were individuals with sensory or motor impairments, uncontrolled chronic diseases, severe psychiatric disorders, clinical signs or neuroimaging findings suggesting vascular disease, and a diagnosis of dementia. All participants underwent an initial (baseline) assessment plus four cognitive and psychosocial reevaluations, performed every six months. All assessments were conducted by duly trained gerontologists and neuropsychologists.

In the present study, participants completed a protocol consisting of a sociodemographic questionnaire to update personal data, and three scales assessing SWB: the General Satisfaction with Life (SGV)^21,28^, which measures satisfaction on a scale of 1-10; Domain-Referenced Life Satisfaction (SVRD), which measures dimensions, such as health, physical capacity, mental capacity, and social involvement on a 5-point scale; and the Positive and Negative Affect Scale, which measures the intensity of affects on a scale from 1 to 5^29^.

The correlations between the collected data and the scores on the cognitive assessments (Addenbrooke Cognitive Examination — Revised — ACE-R^30^ and MMSE^24^) at the end of the intervention (T3) were analyzed. Statistical analyses were performed in three stages. First, the data were entered and the databases were consolidated. Finally, the data relating to SWB, cognitive performance, and sociodemographic variables were also correlated with each other using a heat map.

### Ethical aspects

The study was submitted to the Ethics Committee for Research Involving Humans of the Hospital das Clínicas da Faculdade de Medicina da Universidade de São Paulo (HC-FMUSP), and approved on the 23^rd^ of October 2020, under permit no. 4.357.429.

## RESULTS

Sociodemographic information for 183 participants was collected and analyzed (Table 1). The sample comprised predominantly women. With regard to self-declared color/race, most participants identified as White, followed by Yellow, Brown and Black. Participants had a mean age of 67.32 years, and mean education of 17.03 years of formal study. Regarding marital status, the majority of participants were married. With respect to employment status, most individuals were either retired or pensioners.

**Table 1:** Sociodemographic data (n=183).

Variable	n	%
Sex		
Female	142	77.60
Male	41	22.40
Age		
Mean (SD)	67.32 (±4.88)	
Median (Min.–Max.)	67.00 (60.00–84.00)	
Education		
Mean (SD)	17.03 (±4.81)	
Median (Min.–Max.)	16.00 (8.00–45.00)	
Marital status		
Widowed	24	13.11
Married/Civil union	87	47.54
Single	44	24.04
Separated/Divorced	28	15.30
Retired		
Yes	156	85.25
No	27	14.75
Color		
Black	9	4.92
White	134	73.22
Yellow	21	11.48
Brown	19	10.38

Abbreviation: SD, standard deviation.

Data on cognition and psychosocial variables are presented in Table 2. For cognitive performance, participants attained a mean total score on the ACE-R (assessing cognitive domains) of 92.62, and had an overall satisfactory performance on attention and orientation (mean=17.21), memory (mean=24.10), verbal fluency (mean=11.64), language (mean=25.05) and visuospatial ability (mean=14.57). On the MMSE (assessing overall cognition), participants had a mean score of 28.54, indicating good overall cognitive performance for the sample.

**Table 2:** Data for cognition and psychosocial variables.

Variables	Mean	SD	Median	Minimum	Maximum
ACE-R	92.62	4.79	93.00	73.00	100.00
MMSE	28.54	1.56	29.00	23.00	30.00
Attention and Orientation	17.21	1.17	18.00	12.00	18.00
Memory	24.15	2.23	25.00	16.00	26.00
Fluency	11.64	1.67	12.00	5.00	14.00
Language	25.05	1.18	25.00	18.00	26.00
Visuospatial	14.57	1.58	15.00	8.00	16.00
SVRD Total	4.17	0.51	4.25	2.25	5.00
SVRD Health	4.16	0.65	4.00	1.00	5.00
SVRD Physical_C	4.11	0.70	4.00	1.00	5.00
SVRD Mental_C	4.38	0.62	4.50	2.00	5.00
SVRD Social_I	4.01	0.80	4.00	1.00	5.00
Positive Affect Total	4.08	0.60	4.00	1.67	5.00
Negative Affect Total	1.78	0.60	1.75	1.00	4.25
Gen. Life Sat. Total	8.21	1.43	8.00	2.00	10.00

Abbreviation: SD, standard deviation.

In relation to psychosocial variables, participants had a mean score on the SVRD of 4.17. Of the specific domains assessed, mental capacity had the highest mean satisfaction (4.38), whereas social involvement had the lowest (4.01). Mean scores for health and physical capacity were 4.16 and 4.11, respectively.

Affects were measured using the Positive and Negative Affect scale: the mean score for total positive affect was 4.08 (scale 1-5), while the mean score for total negative affect was relatively low at 1.78, indicating few negative affects. Lastly, scores on the SGV (scale 1–10) showed a robust mean of 8.21, with most participants rating their life at 8.00 (median), and as high as 10.00, suggesting a high level of overall SWB in the sample studied.

**Figure 1:**
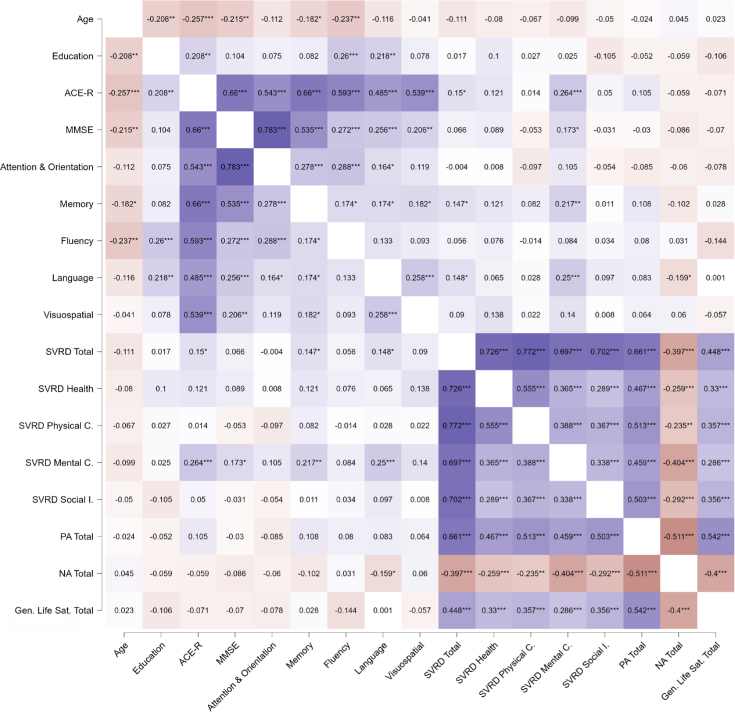
Spearman’s correlation heatmap.

Note: *p<0.05; **p<0.01; ***p<0.001.

A correlation was identified between 24-month SGV and change in cognitive performance. A moderate positive correlation was observed between SGV and MMSE at T3, showing that participants with more marked improvement in cognitive performance also had higher levels of satisfaction. A positive association was also found between SGV and the attention and orientation domains (Figure 1).

In summary, there was a significant improvement in general cognitive performance (ACE-R, p<0.001), memory (p<0.001), verbal fluency (p<0.001), and attention and orientation (p=0.004) after 24 months. However, SGV scores showed a significant positive correlation only with performance on the MMSE (rho=0.449, p<0.001) and on the attention and orientation test of the ACE-R (rho=0.341, p=0.008).

Spearman’s correlation matrix, providing an analysis of the relationships between sociodemographic, cognitive, and psychosocial variables of participants, is presented in Table 3. Age exhibited a significant negative correlation with education and with overall cognitive performance, suggesting that advancing age is associated with less educational attainment and with a decline in cognitive performance. Specifically, for the domains of the ACE-R, age showed a significant negative correlation with memory (rho=-0.182; p=0.013) and verbal fluency (rho=-0.237; p=0.001).

**Table 3:** Spearman’s correlation matrix.

Variable	Spearman	1	2	3	4	5	6	7	8	9	10	11	12	13	14	15	16	17
1. Age	rho	—																
	p-value	—																
2. Education	rho	-0.208	—															
	p-value	0.005	—															
3. ACE-R	rho	-0.257	0.208	—														
	p-value	< .001	0.005	—														
4. MMSE	rho	-0.215	0.104	0.660	—													
	p-value	0.003	0.162	<0.001	—													
5. Attention & Orientation	rho	-0.112	0.075	0.543	0.783	—												
	p-value	0.131	0.311	<0.001	<0.001	—												
6. Memory	rho	-0.182	0.082	0.660	0.535	0.278	—											
	p-value	0.013	0.271	<0.001	<0.001	<0.001	—											
7. Fluency	rho	-0.237	0.260	0.593	0.272	0.288	0.174	—										
	p-value	0.001	<0.001	<0.001	<0.001	<0.001	0.018	—										
8. Language	rho	-0.116	0.218	0.485	0.256	0.164	0.174	0.133	—									
	p-value	0.119	0.003	<0.001	<0.001	0.026	0.018	0.073	—									
9. Visuospatial	rho	-0.041	0.078	0.539	0.206	0.119	0.182	0.093	0.258	—								
	p-value	0.578	0.296	<0.001	0.005	0.108	0.014	0.212	<0.001	—								
10. SVRD Total	rho	-0.111	0.017	0.150	0.066	-0.004	0.147	0.056	0.148	0.090	—							
	p-value	0.133	0.822	0.043	0.376	0.956	0.047	0.452	0.045	0.228	—							
11. SVRD Health	rho	-0.080	0.100	0.121	0.089	0.008	0.121	0.076	0.065	0.138	0.726	—						
	p-value	0.279	0.178	0.103	0.231	0.910	0.102	0.304	0.384	0.062	<0.001	—						
12. SVRD Physical C.	rho	-0.067	0.027	0.014	-0.053	-0.097	0.082	-0.014	0.028	0.022	0.772	0.555	—					
	p-value	0.371	0.719	0.848	0.472	0.194	0.271	0.853	0.704	0.766	<0.001	<0.001	—					
13. SVRD Mental C.	rho	-0.099	0.025	0.264	0.173	0.105	0.217	0.084	0.250	0.140	0.697	0.365	0.388	—				
	p-value	0.181	0.737	<0.001	0.019	0.159	0.003	0.259	<0.001	0.059	<0.001	<0.001	<0.001	—				
14. SVRD Social I.	rho	-0.050	-0.105	0.050	-0.031	-0.054	0.011	0.034	0.097	0.008	0.702	0.289	0.367	0.338	—			
	p-value	0.503	0.157	0.502	0.678	0.467	0.878	0.644	0.191	0.917	<0.001	<0.001	<0.001	<0.001	—			
15. PA Total	rho	-0.024	-0.052	0.105	-0.030	-0.085	0.108	0.080	0.083	0.064	0.661	0.467	0.513	0.459	0.503	—		
	p-value	0.751	0.487	0.156	0.683	0.255	0.145	0.283	0.263	0.390	<0.001	<0.001	<0.001	<0.001	<0.001	—		
16. NA Total	rho	0.045	-0.059	-0.059	-0.086	-0.060	-0.102	0.031	-0.159	0.060	-0.397	-0.259	-0.235	-0.404	-0.292	-0.511	—	
	p-value	0.546	0.430	0.426	0.246	0.424	0.171	0.679	0.032	0.416	<0.001	<0.001	0.001	<0.001	<0.001	<0.001	—	
17. Gen. Life Sat. Total	rho	0.023	-0.106	-0.071	-0.070	-0.078	0.028	-0.144	0.001	-0.057	0.448	0.330	0.357	0.286	0.356	0.542	-0.400	—
	p-value	0.753	0.154	0.342	0.345	0.294	0.708	0.052	0.988	0.446	<0.001	<0.001	<0.001	<0.001	<0.001	<0.001	<0.001	—

By contrast, education showed a significant positive correlation with performance on the ACE-R (rho=0,208; p=0,005), and also with fluency and language domains, suggesting that more years of formal study are associated with better cognitive performance in these areas.

With regard to SWB, domain-referenced life satisfaction (SVRD_Total) showed a significant positive correlation with ACE-R. More specifically, SVRD_C_Mental (satisfaction with mental capacity) was positively correlated with performance on both the ACE-R and MMSE. Hence, this indicates a relationship of satisfaction with mental capacity and cognitive performance. Moreover, total positive affect (PA_Total) exhibited a highly significant positive correlation with SVRD_Total and all its specific domains. As expected, total negative mood (NA_Total) showed a highly significant negative correlation with SVRD_Total and all domains of the SVRD, given that a negative mood is associated with lower life satisfaction. Lastly, SGV showed a highly significant positive correlation with SVRD_Total and its domains, besides a strong positive correlation with total positive mood and a significant negative correlation with total negative mood. Interestingly, the measures of cognition (ACE-R and MMSE) showed no significant correlations with positive or negative moods, suggesting that mood, unlike life satisfaction, was not directly associated with cognitive performance in this sample.

## DISCUSSION

In the present study, the descriptive analysis revealed that the sample predominantly comprised women, which is consistent with the Brazilian demographic profile. The larger proportion of women in the older population can be explained by the phenomenon of feminization of aging, characterized by the predominance of older women in sociocultural activities^31^. This trend stems largely from the longer life expectancy among women, as evidenced by national and international demographic data^32^.

Education was a factor exerting a major influence on cognitive performance, as widely reported in the literature. Lövdén et al.^33^ showed that high-educated individuals tend to have a better performance in functions such as working memory, processing speed, logical reasoning, among others. The results of the present study corroborate these findings: the mean education of participants was 17.03 years, characterizing a group with high educational level and high cognitive performance, as demonstrated by scores on the ACE-R and MMSE.

It is also imperative to consider these findings through the lens of the Social Determinants of Health (SDOH). Recent evidence from a large cohort study indicates that, despite often having stronger social support, women’s cognitive capacity can be disproportionately impacted by adverse SDOH compared to men^34^. However, the present study’s sample is characterized by a significant socioeconomic advantage, evidenced by the high mean years of schooling. This high educational attainment likely serves as a robust protective factor, mitigating the vulnerability to adverse SDOH typically observed in the older female population. Therefore, the ‘ideal’ cognitive performance and high subjective well-being observed here may reflect how socioeconomic privilege buffers against cognitive decline, reinforcing the protective role of education in the intersection between gender and cognitive aging.

Furthermore, having more years of formal education is positively associated with socioeconomic status, favoring greater access to health resources, increased engagement in social and cognitive activities, as well as a greater disposition for adopting healthy habits and avoiding harmful behaviors. According to Bertola and Kochhann^35^, these factors directly influence SWB, and contribute to the development of cognitive reserve, defined as the brain’s ability to offset the negative effects of aging or neurodegenerative diseases, delaying the onset of clinical symptoms^36^.

The current study sample is part of a randomized clinical trial involving cognitive stimulation, a factor which should be taken into account, in that significant gains have been observed in cognitively healthy older adults after participating in structured cognitive intervention programs. For example, the study by Phanasathit et al.^37^, comparing experimental and control groups, found improvements in overall cognition among participants receiving the intervention, which supports the findings of the present study.

Similarly, Sieddecki et al.^38^ reported that high scores on the ACE-R and Satisfaction With Life Scale were associated with higher cognitive functioning, enabling individuals to remain engaged in everyday activities. These authors also showed that better health status was associated with higher levels of life satisfaction, a relationship corroborated by the present study, in which average scores for health and physical capacity domains exceeded 4. Additionally, Patel et al.^39^ found that older adults with poor self-rated health were more likely to have low SWB compared to their counterparts.

Falzarano et al.^40^ carried out a correlations analysis, showing a positive association between life satisfaction and the majority of cognitive variables. This relationship was echoed by findings of the present study, where SVRD_Total exhibited a significant positive correlation with ACE-R. More specifically, SVRD_C_Mental (satisfaction with mental capacity) correlated positively with performance on both the ACE-R and MMSE. In short, older adults with high levels of SWB tend to score higher on these cognitive measures.

However, no significant correlations were found between measures of positive or negative affect and cognitive performance. This lack of association may be partially explained by the characteristics of the sample, composed of cognitively healthy and highly educated older adults, which tends to restrict affective and cognitive variability. Similar findings were reported by Pereira et al.^41^, showing that superior cognition was more closely linked to perceived health and satisfaction than to affective states. In addition, affective measures such as positive and negative affect are more situational and transient, reflecting emotional states sensitive to daily fluctuations rather than stable cognitive-emotional patterns. Studies by Guevarra et al.^42^ and MA et al.^43^ indicate that negative affect predicts short-term cognitive lapses, whereas positive affect demonstrates inconsistent associations across individuals, suggesting that these effects may dissipate in high-functioning populations. In this sense, affective stability and emotional regulation, as explained by socioemotional selectivity theory, may buffer potential interactions between mood and cognition in older adults with high cognitive reserve.

Conversely, life satisfaction represents a more enduring cognitive-evaluative component of well-being, reflecting a stable self-perception that integrates both emotional and cognitive regulation processes. Komalasari et al.^44^ demonstrated that positive attitudes toward aging and higher subjective well-being were associated with lower subjective cognitive decline, suggesting that satisfaction functions as a protective mechanism. Similarly, Hittner et al.^45^ showed that positive affect predicted reduced memory decline only in longitudinal contexts, emphasizing the importance of temporal dynamics. Broader constructs such as cognitive reserve, positive aging attitudes, and engagement in health-promoting behaviors have also been linked to better cognitive outcomes^46,47^. Thus, satisfaction may operate as a consolidated self-perceptual mechanism that mitigates cognitive and functional declines through compensatory behaviors, social engagement, and greater health care^41,48^.

Cachioni et al.^21^ noted that the results of the English Longitudinal Study of Ageing (ELSA) show a tendency for older individuals to have higher levels of SWB compared to middle-aged adults. Consistent with these results, the present study findings revealed that participants had satisfactory levels of positive affects. Taken together, these results suggest that higher levels of well-being may be related to favorable cognitive outcomes in healthy older adults. However, some methodological aspects should be considered when interpreting these findings. The predominance of women and the high educational level of the sample limit the generalization of the results. In addition, the cross-sectional design and participant involvement in the clinical trial may have introduced selection bias, hampering the establishment of causal inferences.

Future studies involving more diverse samples in terms of gender, education, and socioeconomic status, and incorporating longitudinal designs to investigate the causal relationship between subjective well-being and cognition, are warranted. The inclusion of qualitative measures of life experiences and social support could also help elucidate the factors promoting cognitive health in older adults.

In conclusion, the present study revealed that high-educated healthy older adults had high subjective well-being and satisfactory cognitive performance, corroborating the literature on cognitive reserve and social engagement.

## Data Availability

The datasets generated and/or analyzed during the current study are available from the corresponding author upon reasonable request.
